# Clinical Relevance of Urine Flow Rate and Exposure to Polycyclic Aromatic Hydrocarbons

**DOI:** 10.3390/ijerph18105372

**Published:** 2021-05-18

**Authors:** Po-Hsuan Jeng, Tien-Ru Huang, Chung-Ching Wang, Wei-Liang Chen

**Affiliations:** 1 Department of General Medicine, School of Medicine, Tri-Service General Hospital, National Defense Medical Center, Taipei 114, Taiwan; bigsmalleye124@gmail.com (P.-H.J.); sunnysandy1019@gmail.com (T.-R.H.); 2 Department of Surgery, Division of Urology, Tri-Service General Hospital, Taipei 114, Taiwan; 3 Department of Otolaryngology-Head and Neck Surgery, Tri-Service General Hospital, Taipei 114, Taiwan; 4 Department of Family and Community Medicine, Division of Family Medicine, School of Medicine, Tri-Service General Hospital, National Defense Medical Center, Taipei 114, Taiwan; bigching@gmail.com; 5 Department of Family and Community Medicine, Division of Geriatric Medicine, School of Medicine, Tri-Service General Hospital, National Defense Medical Center, Taipei 114, Taiwan

**Keywords:** polycyclic aromatic hydrocarbons, urine flow rate, bladder dysfunction, cross-sectional data, National Health and Nutrition Examination Survey

## Abstract

Background: Polycyclic aromatic hydrocarbon (PAH) metabolites have received increasing attention because several of these organic substances are highly carcinogenic or mutagenic. Exposure to PAHs is associated with many harmful health effects; however, we are not aware of any study that has explored the exposure to PAHs and urinary conditions in the general population. The present work aimed to investigate the correlation among PAH and urine flow rate (UFR). Method: Cross-sectional data from the National Health and Nutrition Examination Survey (NHANES) 2009–2012 were used in our study. A total of 4172 participants and a total of nine PAH metabolites were examined. The UFR was measured as the amount of urine excreted in a period of time (mL/h). Several covariates were adjusted in linear regression models. Result: After adjusting for variables, the PAH metabolites in urine showed a significant correlation with UFR. Dose-dependent associations between PAH metabolites in the urine and UFR were also found. Higher quartiles of PAH metabolites in urine exhibited higher regression coefficients. Conclusion: Our study highlighted that PAH metabolites in urine had a strong association with decreased UFR in the US adult population. These findings support the possibility that PAH exposure is related to bladder dysfunction. Further prospective studies are warranted.

## 1. Introduction

Polycyclic aromatic hydrocarbons (PAHs) are nonpolar molecules that are composed of more than two aromatic rings. PAHs are formed through incomplete combustion from crude oil, wildfire, cigarette smoke and various industrial activities [1]. People can be exposed to PAHs via breathing them in, absorption by the skin, and oral consumption. The great majority of PAHs are excreted from the body through urination [2]. Over the last few years, PAHs have received increasing awareness due to several of these organic substances being highly carcinogenic or mutagenic [3,4]. Benzo(a)pyrene (BaP) causes a DNA adduct, which has been associated with lung cancer [5]. The International Agency for Research on Cancer (IARC) classified BaP as carcinogenic to humans (Group 1) [6] and classified naphthalene as possibly carcinogenic to humans (Group 2B). Although many PAHs, including fluorene and phenanthrene, are undetermined due to a lack of investigations, exposure to PAHs brings out many other potentially harmful effects on human health [7], including oxidative stress [8,9], inflammation [10,11], cardiovascular disease [12,13], respiratory disease [14], and poor fetal development [15]. A cross-sectional study revealed that PAH metabolites, such as 2-hydroxyfluorene, 3-hydroxyfluorene, and 3-hydroxyphenanthrene, have a possible effect on rheumatoid arthritis [16]. Moreover, a recent study demonstrated that total urinary PAH metabolites were dose-dependently associated with increased risk of atherosclerotic cardiovascular disease [17]. In fact, there are 16 PAHs listed on the priority pollutant list of the United States Environmental Protection Agency (US EPA) that need strict monitoring [18]. The short-term effects of PAHs on human health have been revealed to be exacerbated lung function [19] and thrombotic events in people who had coronary heart disease [20]. The long-term effects of PAH exposure were identified as the cause of multiple cancers, such as lung cancer [21], bladder cancer [22], and oral cancers [23]. However, we are not aware of any study that has investigated the possible relevance of exposure to PAHs and urinary conditions in the general population.

The number of studies involving urine biomonitoring for chemical exposure assessment has increased rapidly. In the past, urinary concentrations were considered to represent the level of chemical exposure. For biomonitoring of PAHs, 1-hydroxypyrene is most commonly used as biomarker of exposure to PAHs [24]. However, due to the fact that humans are exposed to a mixture of PAHs, the biomonitoring of other urinary hydroxylated PAHs would give us a more comprehensive assessment of PAH exposure [25]. In fact, the metabolites of urinary PAHs, including naphthalene, fluorene, phenanthrene, and pyrene, are commonly presented as indicators of exposure to PAHs in several research [26,27]. Nevertheless, the random variation, including urine flow rate (UFR), may confound the interpretation of the association between urinary concentration and health outcomes. Thus, studies that depend on spot urine samples should consider collecting additional UFR data [28]. The National Health and Nutrition Examination Survey (NHANES) begun collecting data on UFR in 2009. UFR is measured with uroflowmetry, which is a noninvasive and convenient way to reflect the hydration status and voiding condition [29]. The UFR is regulated by the strength of detrusor contraction and the resistance of the bladder outlet, and it appears to be a useful tool for excluding the presence of obstructive uropathy disease in humans [30,31]. 

Even though many researchers have shown that a variety of PAHs metabolites influences the health status, little awareness has been given to the disclosure of the correlation between exposure to PAHs and bladder function. The previous research has focused on the causation of PAHs and bladder cancer. To date, no previous literature has discussed the influence of PAH exposure to UFR. Thus, the purpose of our analysis was to attempt to find the relationship between PAHs and UFR by analyzing the NHANES dataset of 2009–2012.

## 2. Materials and Methods

### 2.1. Study Population and Design

A total of 20,293 participants from the NHANES dataset of 2009–2012 were involved in our study. NHANES is a continuing survey with a cross-sectional design. Noninstitutionalized citizens in the USA are examined every year, and NHANES is performed by the National Center for Health Statistics (NCHS). NHANES mainly consists of two parts. The first part is interviews with participants at their homes. After agreement was provided by the NCHS Ethics Review Board, every participant completed informed consent forms and completed detailed questionnaires. In the second part, NHANES conducted examinations, including laboratory analysis of biologic samples (blood, urine and tissue samples). We excluded the participants with loss of data, such as biochemistry data, and so finally 4172 participants were included in our study (Table 1). All the data we analyzed in this study were obtained from the website https://www.cdc.gov/nchs/nhanes/index.htm (the NHANES dataset., accessed on 17 May 2021), including the urinary polycyclic aromatic hydrocarbon metabolites, urine flow rate, and relevant covariates.

### 2.2. Measurement and Analysis of Polycyclic Aromatic Hydrocarbons 

Urine samples were collected from the study participants in mobile exam centers (MECs) and stored at −20 °C by trained professionals. Samples with visible contamination with bacteria or mold were rejected. A 0.2 mL sample is the minimum volume for the test. The methodological procedures included enzymatic hydrolysis of glucuronidated/sulfated urinary hydroxylated PAH metabolites, extraction using on-line solid phase extraction, and separation and quantification using the method of isotope dilution high-performance liquid chromatography–tandem mass spectrometry (HPLC/MS/MS). The limit of detection for 1-hydroxynaphthalene is 48 pg/mL; for 2-hydroxynaphthalene it is 40 pg/mL; and for the other OH-PAHs it is 10 pg/mL. The coefficient of variation for each PAH metabolite was as follows: 2.8 to 4.9 for 1-hydroxynaphthalene; 2.4 to 6.3 for 2-hydroxynaphthalene; 4.5 to 8.5 for 3-hydroxyfluorene; 2.5 to 6.2 for 2-hydroxyfluorene; 0.7 to 5.8 for 3-hydroxyphenanthrene; 1.2 to 6.6 for 1-hydroxyphenanthrene; 1.1 to 4.3 for 2-hydroxynaphthalene; 5.0 to 11.7 for 1-hydroxypyrene; and 2.9 to 8.3 for 9-hydroxyfluorene. The summary statistics and quality control chart of each OH-PAH and the whole procedure was described in detail on the webpage of NHANES, Lab Methods for Monohydroxy-Polycyclic Aromatic Hydrocarbons (https://wwwn.cdc.gov/nchs/nhanes/2011–2012/PAH_G.htm, accessed on 17 May 2021). In this study, nine hydroxylated PAH metabolites were examined in total, including 1-hydroxynaphthalene, 2-hydroxynaphthalene, 1-hydroxyphenanthrene, 2-hydroxyphenanthrene, 3-hydroxyphenanthrene, 2-hydroxyfluorene, 3-hydroxyfluorene, 9-hydroxyfluorene, and 1-hydroxypyrene.

### 2.3. Assessment of Urine Flow Rate 

Using uroflowmetry, UFR was measured as the amount of urine excreted in a period of time (mL/h). The study participants had to document their last time of urination before they came to the MECs. Then, they voided at the MECs where their urine amount and time of collection were recorded. The participants’ urine was collected more than two times if their urine volume was not sufficient for analysis. The procedures regarding the urine collection and management were recorded in the NHANES Laboratory/Medical Technologists Procedures Manual (LPM).

### 2.4. Assessment of Covariates

Multivariable adjustment of demographic information, including age, sex, race, medical history, and biochemistry results, was performed in the study. Race was divided into Mexican American, other Hispanic, non-Hispanic white, non-Hispanic black, and other. History of heart disease was defined as the occurrence of congestive heart failure, coronary heart disease, angina pectoris, and heart attack. Smoking status was defined as never smoked or smoking at least 100 cigarettes during one’s lifetime. Aspartate aminotransferase (AST) was detected by the DxC800 with the enzymatic rate method; serum plasma glucose was measured by the DxC800 with Beckman Oxygen electrode (glucose oxidase method) from blood samples obtained from participants. The level of creatinine (Cr) was detected by the DxC800 system with the Jaffe rate method (kinetic alkaline picrate) from urine specimens. Standardized principles were used for all of the protocols and were documented on the NHANES webpage https://wwwn.cdc.gov/nchs/nhanes/2011–2012/BIOPRO_G.htm (accessed on 17 May 2021).

### 2.5. Statistical Methodology

Statistical Package for the Social Sciences version 18.0, for Windows (SPSS Inc., Chicago, IL, USA), was used in our study for analyses. Statistical significance was defined as a *p*-value < 0.001. We used four models to adjust for the following variables: age, sex, race, history of heart disease and smoking, plasma glucose, aspartate aminotransferase, and creatinine. We defined Model 1 as the unadjusted model; Model 2 was adjusted for age, sex, and race; Model 3 was adjusted for the same covariates as Model 2 and additionally for the AST level, creatinine level, and serum glucose level; Model 4 was adjusted for the same covariates as Model 3 and further for history of heart disease and smoking. We normalized the distributions of the UFR with log transformation. The relevance of PAH metabolites and log-transformed UFR was analyzed by linear regression. β coefficients were explained as alterations in log-transformed UFR for each increase in the PAH metabolite level. PAH metabolite levels were divided into quartiles to confirm if there was a dose-dependent association between PAH exposure and decreased UFR by linear regression analysis.

## 3. Results

### 3.1. Characteristics of the Study Population

**Figure 1:**
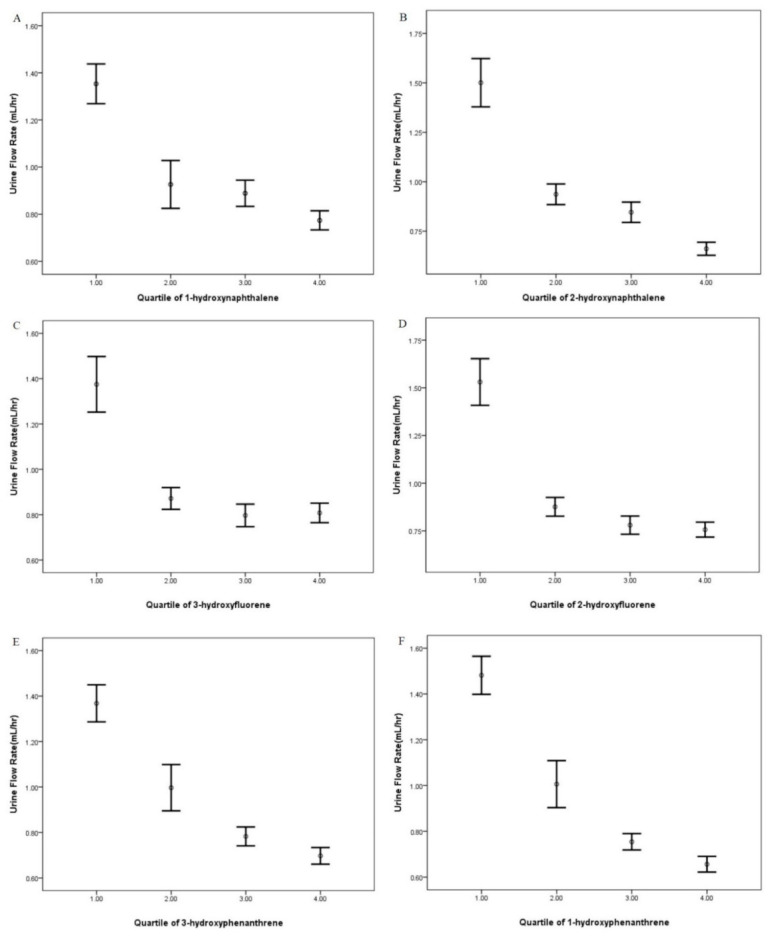

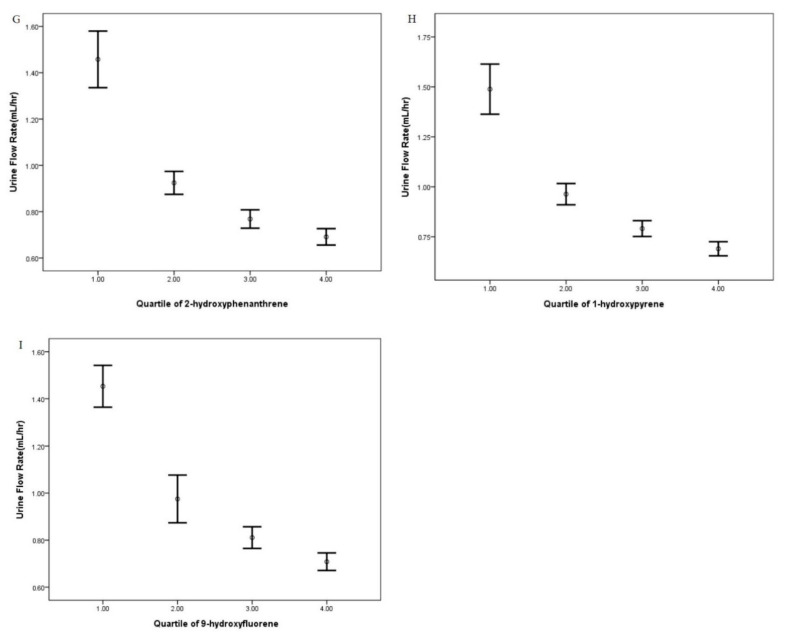
The correlation between quartiles of the PAH metabolites, including (**A**) 1-hydroxynaphthalene, (**B**) 2-hydroxynaphthalene, (**C**) 3-hydroxyfluorene, (**D**) 2-hydroxyfluorene, (**E**) 3-hydroxyphenanthrene, (**F**) 1-hydroxyphenanthrene, (**G**) 2-hydroxyphenanthrene, (**H**) 1-hydroxypyrene, (**I**) 9-hydroxyfluorene, and UFR.

Our study included a total of 4172 people from NHANES from 2009–2012. The characteristics of our study population are listed in Table 1. The average age of the population was 41.64, ranging from 6 to 80 years old. A total of 50.7% of the population was male. The laboratory biochemical levels of aspartate transaminase (U/L), creatinine (mg/dL), and glucose (mg/dL) were 25.90 (ranging from 7 to 733), 0.89 (ranging from 0.16 to 7.33), and 100.23 (ranging from 34 to 458), respectively. Histories of congestive heart failure, coronary heart disease, angina/angina pectoris, and heart attack were reported. The correlation between quartiles of the PAH metabolites in urine and UFR is shown in Figure 1.

### 3.2. Polycyclic Aromatic Hydrocarbon Metabolites and Urine Flow Rate

As presented in Table 2, PAH metabolites in urine showed a significant correlation with UFR. Associations were discovered by linear regression analysis, and all PAH metabolites in urine were associated with decreased UFR except 1-hydroxynaphthalene when unadjusted and even when fully adjusted (all PAH metabolites except 1-hydroxynaphthalene, Models 1–4, *p*-value < 0.001). The β coefficients between PAH metabolites in urine and UFR were as follows: 1-hydroxynaphthalene 0.000 (*p*-value = 0.023, 95% CI = 0–0 which does not contain 0), 0.000 (*p*-value = 0.060, 95% CI = 0–0 which does not contain 0), 0.000 (*p*-value = 0.057, 95% CI = 0–0 which does not contain 0), and 0.000 (*p*-value = 0.061, 95% CI = 0–0 which does not contain 0) for Models 1–4, respectively; for the β coefficients and *p*-values for the other PAH metabolites, please refer to Table 2.

### 3.3. Dose-Dependent Relationship between Polycyclic Aromatic Hydrocarbon Metabolites and Urine Flow Rate

Table 3 presents the dose-dependent effects of the PAH metabolites in urine and UFR. For all PAH metabolites, the *p*-values were <0.001. This indicates that the dose-dependent associations were significant between quartiles of PAH metabolites in urine and UFR. With or without adjustment, higher quartiles of PAH metabolites in urine had higher regression coefficients.

## 4. Discussion

Urinary biomonitoring is an optimal method to assess exposure to numerous chemical elements and metabolites. Because of individual exposure to PAHs through numerous paths, including breathing them in, oral consumption, and absorption by the skin, the amount of PAHs in vivo from the environment is difficult to estimate. By the time the PAHs are absorbed into the body, they are mainly metabolized by cytochrome P450-dependent monooxygenases (CYP1A1, 1A2, 1B1, and 3A4) to hydrosoluble metabolites and excreted in urine [32]. Consequently, the urinary PAH concentration is frequently used as biomarkers to evaluate a population’s current exposure to PAHs [33]. While 24-h urine samples are preferred over spot urine samples, they are impractical for large-scale biomonitoring due to the large amount of the samples and participant compliance issues [34]. Collection of spot urine or first morning voids is an alternative method, but several variables should be appropriately adjusted. NHANES examinations began to collect UFR data in 2009, which entailed collecting urine sample from participants in the Mobile Examination Center (MEC), leading to the evaluation of the total amount of substances excreted in a 24-h period [35]. Previous research has shown the dose-dependence between urinary concentrations of PAH metabolites and the risk of bladder cancer [22]. However, our present work is the foremost study to attempt to describe the correlation between PAH metabolites and UFR. 

This study elucidates the strong relevance between PAH metabolites in urine and UFR in multiple models. In addition, a dose-dependent relationship between PAH metabolites in urine and UFR was found. In fact, no literature on the relationship between PAH metabolites and UFR has been found to date. 

A number of studies have indicated that PAH metabolites may lead to oxidative stress and inflammation [8,9,10,11,36]. Several studies have shown that PAHs are ligands of aryl hydrocarbon receptors (AhR), and are able to activate these receptors [37,38]. AhR is a ligand-activated transcription factor that is located in the cytoplasm. Upon ligand binding, the AhR complex translocates to the nucleus and a part of the AhR complex is dissociated in the cytoplasm to stimulate c-Src activity, which is followed by activation of mitogen-activated protein kinase signaling [39], contributing to induce the expression of cyclooxygenase-2 (COX-2) and NADPH oxidase-2 (NOX-2), which increased reactive oxygen species [40]. The representative PAHs of this possible effect is high molecular weight PAHs, including BaP, benzo[k]fluoranthene, and indeno [1,2,3-cd]pyrene [41]. Exposure to PAHs is positively associated with increasing levels of C-reactive protein and leukocyte counts. In an exploratory data of 200 pregnant women, 2-hydroxynapthalene, 9-hydroxyphenanthrene, and 1-hydroxypyrene were found to have a significant association to the inflammation markers [42]. In animal models, the importance of oxidative stress has been investigated in many studies and correlated with bladder dysfunction [43,44]. Oxidative stress causes damage to both the bladder mucosa and muscular layer; moreover, functional bladder disorders develop [45]. Oxidative stress probably underlies the relationship between PAH metabolites and UFR.

Another possible explanation of this correlation may be atherosclerosis. Numerous studies have reported that PAHs may deteriorate blood vessel conditions by causing atherosclerosis [20,46,47]. A cross sectional study revealed that high levels of naphthalene, fluorene, and 2-phenanthrene increased the risk of dyslipidemia [46]. In addition, another recent study demonstrated that total urinary PAH metabolites were dose-dependent associated with increased risk of atherosclerotic cardiovascular disease [17]. In prior investigation, 2-hydroxynaphthalene, 2-hydroxyfluorene, 1-hydroxypyrene, and 1-hydroxynaphthalene tend to exhibit estrogenic activity [47], which could induce adipocyte-fatty acid binding protein (AFABP) released from adipocytes. The influence of AFABP on atherosclerosis is based on their effect on macrophages [48]. Lipid metabolism in macrophages is changed by AFABP, which facilitates the formation of foam cells and develops atherosclerosis [49]. Research on human subjects revealed that a genetic variation at the AFABP locus contributes to decreased adipose tissue AFABP expression as well as lower serum triglyceride levels [50]. In this aspect, adipocyte–fatty acid-binding protein was recently regarded as a biomarker to predict cardiovascular disease and the progression of atherosclerosis [51]. In the past few years, a causation between atherosclerosis and bladder dysfunction has been identified [52,53]. The vesical arteries provide the blood supply of the bladder, and atherosclerotic change in the arteries may influence the blood flow of the bladder, which has a close relationship with bladder wall compliance [54]. The correlation between these conditions suggests that exposure to PAHs may be responsible for bladder dysfunction.

Finally, neurotoxicity of PAH metabolites has been explored in many studies [55,56]. BaP induced neurotoxicity and its mode of action was found in a rodent research [57]. The potential pathway may be the anti-acetylcholinesterase activity [58,59,60]. As far as we know, cholinergic transmission is the major excitatory mechanism in the human bladder [61]. Neurologic impairment may be responsible for interfering with bladder smooth muscle cells, contributing to decreased UFR.

As mentioned above, these findings might indicate possible mechanisms underlying the association between PAH metabolites and UFR in our study. Dose-dependent associations between PAH metabolites in urine and UFR were observed in our study. As we are aware, this is the foremost study to demonstrate the dose-dependent effects of PAH metabolites on UFR.

Several limitations should be mentioned in our study. The major concern is the study design of NHANES is cross-sectional, preventing the determination of a causal relationship between PAH metabolites and decreased UFR. Additionally, no assessment of detrusor condition by cystometry was included in the dataset, so we cannot precisely infer the influence of PAHs on the bladder. Another important limitation is that PAH metabolites were examined for each participant using only one spot urine sample, which may not provide enough information about long-term exposure to PAHs [62]. Finally, the medical history of the patient may be influenced by recall bias.

## 5. Conclusions

Our study highlighted that PAH metabolites in urine had a strong association with decreased UFR in the US adult population. In addition, dose-dependent effects were also observed in our study. These findings support the possibility that PAH exposure is related to bladder dysfunction. Further prospective studies on the association and the causes of the association between PAH exposure and decreased UFR are warranted. Recognizing the mechanism may be helpful to prevent or treat the toxicity related to PAH exposure.

## Figures and Tables

**Table 1:** Characteristics of the study population (*n* = 4172).

Variables	Percent or Mean (Range)
**Continuous Variables, Mean (Range)**	
Age (years)	41.64 (6–80)
Aspartate transaminase (U/L)	25.90(7–733)
Creatinine (mg/dL)	0.89(0.16–7.33)
Glucose (mg/dL)	100.23(34–458)
1-hydroxynaphthalene (ng/mL)	26.47(0.05–16658.08)
2-hydroxynaphthalene (ng/mL)	7.94(0.13–181.35)
3-hydroxyfluorene (ng/mL)	0.27(0.01–6.39)
2-hydroxyfluorene (ng/mL)	0.57(0.01–15.07)
3-hydroxyphenanthrene (ng/mL)	0.12(0.01–6.68)
1-hydroxyphenanthrene (ng/mL)	0.20(0.01–7.48)
2-hydroxyphenanthrene (ng/mL)	0.10(0.01–3.26)
1-hydroxypyrene (ng/mL)	0.22(0.01–8.52)
9-hydroxyfluorene (ng/mL)	0.52(0.01–32.55)
**Category Variables, (%)**	
Gender	
Male	50.7
Female	49.3
Race	
Mexican American	15.0
Other Hispanic	10.7
Non-Hispanic White	41.8
Non-Hispanic Black	21.4
Other	11.0
Congestive heart failure	2.5
Coronary heart disease	3.4
Angina/angina pectoris	1.6
Heart attack	3.2
Ever smoking	36.8

**Table 2:** Associations between the PAH metabolites and urine flow rate.

Variables	Model 1 ^a^ Β ^b^ (95% CI)	*p*-Value	Model 2 ^a^ Β ^b^ (95% CI)	*p*-Value	Model 3 Β ^b^ (95% CI)	*p*-Value	Model 4 Β ^b^ (95% CI)	*p*-Value
1-hydroxynaphthalene	0.000 (0.000, 0.000)	0.023	0.000 (0.000, 0.000)	0.060	0.000 (0.000, 0.000)	0.057	0.000 (0.000, 0.000)	0.061
2-hydroxynaphthalene	−0.017 (−0.020, −0.014)	<0.001	−0.018 (−0.021, −0.016)	<0.001	−0.018 (−0.021, −0.015)	<0.001	−0.019 (−0.021, −0.016)	<0.001
3-hydroxyfluorene	−0.211 (−0.262, −0.159)	<0.001	−0.248 (−0.299, −0.197)	<0.001	−0.250 (−0.301, −0.199)	<0.001	−0.274 (−0.328, −0.220)	<0.001
2-hydroxyfluorene	−0.128 (−0.156, −0.100)	<0.001	−0.145 (−0.173, −0.117)	<0.001	−0.146 (−0.173, −0.118)	<0.001	−0.159 (−0.188, −0.130)	<0.001
3-hydroxyphenanthrene	−0.527 (−0.666, −0.389)	<0.001	−0.614 (−0.752, −0.477)	<0.001	−0.623 (−0.760, −0.486)	<0.001	−0.630 (−0.769, −0.491)	<0.001
1-hydroxyphenanthrene	−0.545 (−0.648, −0.443)	<0.001	−0.573 (−0.675, −0.472)	<0.001	−0.584 (−0.685, −0.483)	<0.001	−0.586 (−0.687, −0.484)	<0.001
2-hydroxyphenanthrene	−0.871 (−1.070, −0.673)	<0.001	−0.987 (−1.183, −0.791)	<0.001	−1.001(−1.197, −0.806)	<0.001	−1.008 (−1.205, −0.810)	<0.001
1-hydroxypyrene	−0.267 (−0.339, −0.195)	<0.001	−0.310 (−0.381, −0.239)	<0.001	−0.318 (−0.389, −0.247)	<0.001	−0.323 (−0.395, −0.251)	<0.001
9-hydroxyfluorene	−0.068 (−0.092, −0.043)	<0.001	−0.074 (−0.098, −0.050)	<0.001	−0.074 (−0.098, −0.050)	<0.001	−0.074 (−0.098, −0.049)	<0.001

^a^ Adjusted covariates: Model 1: unadjusted; Model 2: Model 1 + age + gender + race; Model 3: Model 2 + AST + Cr + glucose, serum; Model 4: Model 3 + history of heart disease + history of smoking. ^b^ β coefficients were interpreted as the change in the log-transformed urine flow rate for each increase in different PAHs.

**Table 3:** Association between quartiles of the PAH metabolites and urine flow rate.

PAH Metabolites		Q2 vs. Q1		Q3 vs. Q1		Q4 vs. Q1		*p* for Trend
		β (95% CI)	*p*-Value	β (95% CI)	*p*-Value	β (95% CI)	*p*-Value	
1-hydroxynaphthalene (ng/L)	Model 1	−0.379 (−0.461, −0.297)	<0.001	−0.380 (−0.462, −0.298)	<0.001	−0.465 (−0.547, −0.383)	<0.001	<0.001
	Model 2	−0.392 (−0.473, −0.311)	<0.001	−0.386 (−0.466, −0.305)	<0.001	−0.494 (−0.573, −0.413)	<0.001	<0.001
	Model 3	−0.389 (−0.469, −0.308)	<0.001	−0.383 (−0.463, −0.302)	<0.001	−0.494 (−0.575, −0.413)	<0.001	<0.001
	Model 4	−0.389 (−0.469, −0.308)	<0.001	−0.390 (−0.471, −0.309)	<0.001	−0.521 (−0.606, −0.436)	<0.001	<0.001
2-hydroxynaphthalene (ng/L)	Model 1	−0.358 (−0.438, −0.278)	<0.001	−0.509 (−0.589, −0.429)	<0.001	−0.657 (−0.737, −0.577)	<0.001	<0.001
	Model 2	−0.379 (−0.457, −0.300)	<0.001	−0.555 (−0.633, −0.476)	<0.001	−0.716 (−0.796, −0.637)	<0.001	<0.001
	Model 3	−0.378 (−0.456, −0.299)	<0.001	−0.551 (−0.630, −0.473)	<0.001	−0.715 (−0.795, −0.636)	<0.001	<0.001
	Model 4	−0.387 (−0.465, −0.309)	<0.001	−0.569 (−0.648, −0.490)	<0.001	−0.755 (−0.837, −0.673)	<0.001	<0.001
3-Hydroxyfluorene (ng/L)	Model 1	−0.364 (−0.446, −0.283)	<0.001	−0.467 (−0.549, −0.386)	<0.001	−0.425 (−0.507, −0.343)	<0.001	<0.001
	Model 2	−0.396 (−0.475, −0.316)	<0.001	−0.549 (−0.630, −0.469)	<0.001	−0.529 (−0.611, −0.448)	<0.001	<0.001
	Model 3	−0.396 (−0.476, −0.317)	<0.001	−0.553 (−0.633, −0.472)	<0.001	−0.533 (−0.614, −0.452)	<0.001	<0.001
	Model 4	−0.398 (−0.478, −0.319)	<0.001	−0.561 (−0.641, −0.480)	<0.001	−0.582 (−0.669, −0.495)	<0.001	<0.001
2-Hydroxyfluorene (ng/L)	Model 1	−0.460 (−0.539, −0.380)	<0.001	−0.600 (−0.680, −0.519)	<0.001	−0.583 (−0.663, −0.502)	<0.001	<0.001
	Model 2	−0.491 (−0.568, −0.413)	<0.001	−0.678 (−0.756, −0.599)	<0.001	−0.688 (−0.767, −0.608)	<0.001	<0.001
	Model 3	−0.490 (−0.568, −0.413)	<0.001	−0.672 (−0.751, −0.593)	<0.001	−0.687 (−0.767, −0.608)	<0.001	<0.001
	Model 4	−0.494 (−0.571, −0.416)	<0.001	−0.685 (−0.764, −0.606)	<0.001	−0.745 (−0.829, −0.660)	<0.001	<0.001
3-Hydroxyphenanthrene (ng/L)	Model 1	−0.339 (−0.420, −0.259)	<0.001	−0.484 (−0.565, −0.404)	<0.001	−0.579 (−0.661, −0.497)	<0.001	<0.001
	Model 2	−0.370 (−0.448, −0.292)	<0.001	−0.546 (−0.625, −0.467)	<0.001	−0.671 (−0.752, −0.590)	<0.001	<0.001
	Model 3	−0.377 (−0.455, −0.299)	<0.001	−0.551 (−0.630, −0.473)	<0.001	−0.677 (−0.758, −0.597)	<0.001	<0.001
	Model 4	−0.382 (−0.460, −0.304)	<0.001	−0.561 (−0.640, −0.482)	<0.001	−0.707 (−0.790, −0.625)	<0.001	<0.001
1-Hydroxyphenanthrene (ng/L)	Model 1	−0.372 (−0.451, −0.293)	<0.001	−0.546 (−0.625, −0.467)	<0.001	−0.715 (−0.795, −0.636)	<0.001	<0.001
	Model 2	−0.382 (−0.459, −0.304)	<0.001	−0.579 (−0.656, −0.501)	<0.001	−0.756 (−0.834, −0.678)	<0.001	<0.001
	Model 3	−0.386 (−0.463, −0.309)	<0.001	−0.593 (−0.670, −0.516)	<0.001	−0.773 (−0.851, −0.656)	<0.001	<0.001
	Model 4	−0.389 (−0.466, −0.312)	<0.001	−0.600 (−0.677, −0.522)	<0.001	−0.785 (−0.863, −0.706)	<0.001	<0.001
2-Hydroxyphenanthrene (ng/L)	Model 1	−0.364 (−0.444, −0.284)	<0.001	−0.494 (−0.575, −0.412)	<0.001	−0.599 (−0.680, −0.518)	<0.001	<0.001
	Model 2	−0.395 (−0.473, −0.317)	<0.001	−0.561 (−0.641, −0.482)	<0.001	−0.680 (−0.760, −0.600)	<0.001	<0.001
	Model 3	−0.407 (−0.485, −0.329)	<0.001	−0.574 (−0.654, −0.495)	<0.001	−0.696 (−0.776, −0.616)	<0.001	<0.001
	Model 4	−0.411 (−0.489, −0.334)	<0.001	−0.582 (−0.661, −0.502)	<0.001	−0.717 (−0.798, −0.635)	<0.001	<0.001
1-hydroxypyrene (ng/L)	Model 1	−0.301 (−0.382, −0.221)	<0.001	−0.460 (−0.541, −0.380)	<0.001	−0.600 (−0.681, −0.518)	<0.001	<0.001
	Model 2	−0.347 (−0.426, −0.268)	<0.001	−0.553 (−0.632, −0.473)	<0.001	−0.713 (−0.794, −0.632)	<0.001	<0.001
	Model 3	−0.368 (−0.447, −0.289)	<0.001	−0.571 (−0.650, −0.491)	<0.001	−0.733 (−0.814, −0.652)	<0.001	<0.001
	Model 4	−0.370 (−0.449, −0.291)	<0.001	−0.585 (−0.665, −0.505)	<0.001	−0.765 (−0.848, −0.682)	<0.001	<0.001
9-hydroxyfluorene (ng/L)	Model 1	−0.375 (−0.455, −0.294)	<0.001	−0.474 (−0.555, −0.393)	<0.001	−0.596 (−0.677, −0.514)	<0.001	<0.001
	Model 2	−0.390 (−0.469, −0.311)	<0.001	−0.524 (−0.603, −0.444)	<0.001	−0.660 (−0.740, −0.579)	<0.001	<0.001
	Model 3	−0.394 (−0.473, −0.316)	<0.001	−0.521 (−0.600, −0.442)	<0.001	−0.663 (−0.743, −0.583)	<0.001	<0.001
	Model 4	−0.401 (−0.479, −0.322)	<0.001	−0.534 (−0.614, −0.454)	<0.001	−0.689 (−0.771, −0.607)	<0.001	<0.001

Adjusted covariates: Model 1: unadjusted; Model 2: Model 1 + age + gender + race; Model 3: Model 2 + AST + Cr + glucose, serum; Model 4: Model 3 + history of heart disease + history of smoking. Independent variables: 1-hydroxynaphthalene, 2-hydroxynaphthalene, 3-Hydroxyfluorene, 2-Hydroxyfluorene, 3 Hydroxyphenanthrene, 1-Hydroxyphenanthrene, 2-Hydroxyphenanthrene, 1-hydroxypyrene, 9-hydroxyfluoren, and 4-phenanthrene (ng/L). Dependent variables: urine flow rates (log mL/h). Definition of abbreviations: Q1 = Quartile 1, Q2 = Quartile 2, Q3 = Quartile 3, and Q4 = Quartile.

## Data Availability

Publicly available datasets were analyzed in this study. This data can be found here: https://wwwn.cdc.gov/nchs/nhanes/, accessed on 17 May 2021.

## Abbreviations

Aryl hydrocarbon receptors	AhR
Adipocyte-fatty acid binding protein	AFABP
Benzo a pyrene	BaP
Centers for Disease Control	CDC
Cyclooxygenase-2	COX-2
International Agency for Research on Cancer	IARC
National Health and Nutrition Examination Survey	NHANES
National Center for Health Statistics	NCHS
NADPH oxidase-2	NOX-2
Polycyclic aromatic hydrocarbons	PAHs
Urine flow rate	UFR
United States Environmental Protection Agency	US EPA

## References

[1] Wick A.F., Haus N.W., Sukkariyah B.F., Haering K.C., Daniels W.L. (2011). Remediation of PAH-Contaminated Soils and Sediments: A Literature Review.

[2] Campo L., Rossella F., Pavanello S., Mielzynska D., Siwinska E., Kapka L., Bertazzi P.A., Fustinoni S. (2010). Urinary profiles to assess polycyclic aromatic hydrocarbons exposure in coke-oven workers. Toxicol. Lett..

[3] McCarrick S., Cunha V., Zapletal O., Vondráček J., Dreij K. (2019). In vitro and in vivo genotoxicity of oxygenated polycyclic aromatic hydrocarbons. Env. Pollut..

[4] Srogi K. (2007). Monitoring of environmental exposure to polycyclic aromatic hydrocarbons: A review. Environ. Chem. Lett..

[5] Barnes J.L., Zubair M., John K., Poirier M.C., Martin F.L. (2018). Carcinogens and DNA damage. Biochem. Soc. Trans..

[6] IARC Working Group on the Evaluation of Carcinogenic Risks to Humans (2012). Chemical agents and related occupations. IARC Monogr. Eval. Carcinog. Risks. Hum..

[7] Gao P., da Silva E., Hou L., Denslow N.D., Xiang P., Ma L.Q. (2018). Human exposure to polycyclic aromatic hydrocarbons: Metabolomics perspective. Environ. Int..

[8] Bortey-Sam N., Ikenaka Y., Akoto O., Nakayama S.M.M., Asante K.A., Baidoo E., Obirikorang C., Saengtienchai A., Isoda N., Nimako C. (2017). Oxidative stress and respiratory symptoms due to human exposure to polycyclic aromatic hydrocarbons (PAHs) in Kumasi, Ghana. Environ. Pollut..

[9] Liu Y., Zhang H., Zhang H., Niu Y., Fu Y., Nie J., Yang A., Zhao J., Yang J. (2018). Mediation effect of AhR expression between polycyclic aromatic hydrocarbons exposure and oxidative DNA damage among Chinese occupational workers. Environ. Pollut..

[10] Alshaarawy O., Zhu M., Ducatman A., Conway B., Andrew M.E. (2013). Polycyclic aromatic hydrocarbon biomarkers and serum markers of inflammation. A positive association that is more evident in men. Environ. Res..

[11] Andersen M.H.G., Saber A.T., Pedersen J.E., Pedersen P.B., Clausen P.A., Lohr M., Kermanizadeh A., Loft S., Ebbehoj N.E., Hansen A.M. (2018). Assessment of polycyclic aromatic hydrocarbon exposure, lung function, systemic inflammation, and genotoxicity in peripheral blood mononuclear cells from firefighters before and after a work shift. Environ. Mol. Mutagen..

[12] Clark J.D., Serdar B., Lee D.J., Arheart K., Wilkinson J.D., Fleming L.E. (2012). Exposure to polycyclic aromatic hydrocarbons and serum inflammatory markers of cardiovascular disease. Environ. Res..

[13] He X., Chen Y., Zhang C., Gong W., Zhang X., Nie S. (2018). Polycyclic Aromatic Hydrocarbons from Particulate Matter 2.5 (PM2.5) in Polluted Air Changes miRNA Profile Related to Cardiovascular Disease. Med. Sci. Monit..

[14] Miller R.L., Garfinkel R., Horton M., Camann D., Perera F.P., Whyatt R.M., Kinney P.L. (2004). Polycyclic aromatic hydrocarbons, environmental tobacco smoke, and respiratory symptoms in an inner-city birth cohort. Chest.

[15] Drwal E., Rak A., Gregoraszczuk E.L. (2019). Review: Polycyclic aromatic hydrocarbons (PAHs)-Action on placental function and health risks in future life of newborns. Toxicology.

[16] Sun L., Ye Z., Ling Y., Cai S., Xu J., Fan C., Zhong Y., Shen Q., Li Y. (2020). Relationship between polycyclic aromatic hydrocarbons and rheumatoid arthritis in US general population, NHANES 2003–2012. Sci. Total. Environ..

[17] Yang L., Guo W., Zeng D., Ma L., Lai X., Fang Q., Guo H., Zhang X. (2019). Heart rate variability mediates the association between polycyclic aromatic hydrocarbons exposure and atherosclerotic cardiovascular disease risk in coke oven workers. Chemosphere.

[18] Keith L.H. (2015). The Source of U.S. EPA’s Sixteen PAH Priority Pollutants. Polycycl. Aromat. Compd..

[19] Mu G., Fan L., Zhou Y., Liu Y., Ma J., Yang S., Wang B., Xiao L., Ye Z., Shi T. (2019). Personal exposure to PM(2.5)-bound polycyclic aromatic hydrocarbons and lung function alteration: Results of a panel study in China. Sci. Total. Environ..

[20] Holme J.A., Brinchmann B.C., Refsnes M., Lag M., Ovrevik J. (2019). Potential role of polycyclic aromatic hydrocarbons as mediators of cardiovascular effects from combustion particles. Environ. Health.

[21] Moorthy B., Chu C., Carlin D.J. (2015). Polycyclic aromatic hydrocarbons: From metabolism to lung cancer. Toxicol. Sci..

[22] Mastrangelo G., Fadda E., Marzia V. (1996). Polycyclic aromatic hydrocarbons and cancer in man. Environ. Health Perspect..

[23] Kamangar F., Schantz M.M., Abnet C.C., Fagundes R.B., Dawsey S.M. (2008). High levels of carcinogenic polycyclic aromatic hydrocarbons in mate drinks. Cancer Epidemiol. Biomark. Prev..

[24] Huang X., Deng X., Li W., Liu S., Chen Y., Yang B., Liu Q. (2019). Internal exposure levels of polycyclic aromatic hydrocarbons in children and adolescents: A systematic review and meta-analysis. Environ. Health Prev. Med..

[25] Dobraca D., Lum R., Sjödin A., Calafat A.M., Laurent C.A., Kushi L.H., Windham G.C. (2018). Urinary biomarkers of polycyclic aromatic hydrocarbons in pre- and peri-pubertal girls in Northern California, predictors of exposure and temporal variability. Environ. Res..

[26] Urbancova K., Dvorakova D., Gramblicka T., Sram R.J., Hajslova J., Pulkrabova J. (2020). Comparison of polycyclic aromatic hydrocarbon metabolite concentrations in urine of mothers and their newborns. Sci. Total Environ..

[27] Murawski A., Roth A., Schwedler G., Schmied-Tobies M.I.H., Rucic E., Pluym N., Scherer M., Scherer G., Conrad A., Kolossa-Gehring M. (2020). Polycyclic aromatic hydrocarbons (PAH) in urine of children and adolescents in Germany—human biomonitoring results of the German Environmental Survey 2014–2017 (GerES V). Int. J. Hyg. Environ. Health.

[28] Hays S.M., Aylward L.L., Blount B.C. (2015). Variation in urinary flow rates according to demographic characteristics and body mass index in NHANES: Potential confounding of associations between health outcomes and urinary biomarker concentrations. Environ. Health Perspect..

[29] Middleton D.R., Watts M.J., Lark R.M., Milne C.J., Polya D.A. (2016). Assessing urinary flow rate, creatinine, osmolality and other hydration adjustment methods for urinary biomonitoring using NHANES arsenic, iodine, lead and cadmium data. Environ. Health.

[30] Ouslander J.G. (2004). Management of overactive bladder. N. Engl. J. Med..

[31] DuBeau C.E., Yalla S.V., Resnick N.M. (1998). Improving the utility of urine flow rate to exclude outlet obstruction in men with voiding symptoms. J. Am. Geriatr. Soc..

[32] Jacob J., Seidel A. (2002). Biomonitoring of polycyclic aromatic hydrocarbons in human urine. J. Chromatogr. B Analyt. Technol. Biomed. Life. Sci..

[33] Gunier R.B., Reynolds P., Hurley S.E., Yerabati S., Hertz A., Strickland P., Horn-Ross P.L. (2006). Estimating exposure to polycyclic aromatic hydrocarbons: A comparison of survey, biological monitoring, and geographic information system-based methods. Cancer Epidemiol. Biomark. Prev..

[34] Yeh H.C., Lin Y.S., Kuo C.C., Weidemann D., Weaver V., Fadrowski J., Neu A., Navas-Acien A. (2015). Urine osmolality in the US population: Implications for environmental biomonitoring. Environ. Res..

[35] Pfeiffer C.M., Lacher D.A., Schleicher R.L., Johnson C.L., Yetley E.A. (2017). Challenges and Lessons Learned in Generating and Interpreting NHANES Nutritional Biomarker Data. Adv. Nutr..

[36] Everett C.J., King D.E., Player M.S., Matheson E.M., Post R.E., Mainous A.G. (2010). Association of urinary polycyclic aromatic hydrocarbons and serum C-reactive protein. Environ. Res..

[37] Weng C.M., Wang C.H., Lee M.J., He J.R., Huang H.Y., Chao M.W., Chung K.F., Kuo H.P. (2018). Aryl hydrocarbon receptor activation by diesel exhaust particles mediates epithelium-derived cytokines expression in severe allergic asthma. Allergy.

[38] Vogel C.F.A., Kado S.Y., Kobayashi R., Liu X., Wong P., Na K., Durbin T., Okamoto R.A., Kado N.Y. (2019). Inflammatory marker and aryl hydrocarbon receptor-dependent responses in human macrophages exposed to emissions from biodiesel fuels. Chemosphere.

[39] Vogeley C., Esser C., Tüting T., Krutmann J., Haarmann-Stemmann T. (2019). Role of the Aryl Hydrocarbon Receptor in Environmentally Induced Skin Aging and Skin Carcinogenesis. Int. J. Mol. Sci..

[40] Vogel C.F.A., van Winkle L.S., Esser C., Haarmann-Stemmann T. (2020). The aryl hydrocarbon receptor as a target of environmental stressors—Implications for pollution mediated stress and inflammatory responses. Redox. Biol..

[41] Vondráček J., Pěnčíková K., Neča J., Ciganek M., Grycová A., Dvořák Z., Machala M. (2017). Assessment of the aryl hydrocarbon receptor-mediated activities of polycyclic aromatic hydrocarbons in a human cell-based reporter gene assay. Environ. Pollut..

[42] Ferguson K.K., McElrath T.F., Pace G.G., Weller D., Zeng L., Pennathur S., Cantonwine D.E., Meeker J.D. (2017). Urinary Polycyclic Aromatic Hydrocarbon Metabolite Associations with Biomarkers of Inflammation, Angiogenesis, and Oxidative Stress in Pregnant Women. Environ. Sci. Technol..

[43] Nomiya M., Sagawa K., Yazaki J., Takahashi N., Kushida N., Haga N., Aikawa K., Matsui T., Oka M., Fukui T. (2012). Increased bladder activity is associated with elevated oxidative stress markers and proinflammatory cytokines in a rat model of atherosclerosis-induced chronic bladder ischemia. Neurourol. Urodyn..

[44] Sezginer E.K., Yilmaz-Oral D., Lokman U., Nebioglu S., Aktan F., Gur S. (2019). Effects of varying degrees of partial bladder outlet obstruction on urinary bladder function of rats: A novel link to inflammation, oxidative stress and hypoxia. Low. Urin. Tract. Symptoms.

[45] Kirpatovsky V.I., Plotnikov E.Y., Mudraya I.S., Golovanov S.A., Drozhzheva V.V., Khromov R.A., Chernikov D.Y., Skulachev V.P., Zorov D.B. (2013). Role of oxidative stress and mitochondria in onset of urinary bladder dysfunction under acute urine retention. Biochemistry.

[46] Ranjbar M., Rotondi M.A., Ardern C.I., Kuk J.L. (2015). Urinary Biomarkers of Polycyclic Aromatic Hydrocarbons Are Associated with Cardiometabolic Health Risk. PLoS ONE.

[47] Schultz T.W., Sinks G.D. (2002). Xenoestrogenic gene expression: Structural features of active polycyclic aromatic hydrocarbons. Env. Toxicol. Chem..

[48] Makowski L., Boord J.B., Maeda K., Babaev V.R., Uysal K.T., Morgan M.A., Parker R.A., Suttles J., Fazio S., Hotamisligil G.S. (2001). Lack of macrophage fatty-acid-binding protein aP2 protects mice deficient in apolipoprotein E against atherosclerosis. Nat. Med..

[49] Fu Y., Luo L., Luo N., Garvey W.T. (2006). Lipid metabolism mediated by adipocyte lipid binding protein (ALBP/aP2) gene expression in human THP-1 macrophages. Atherosclerosis.

[50] Tuncman G., Erbay E., Hom X., de Vivo I., Campos H., Rimm E.B., Hotamisligil G.S. (2006). A genetic variant at the fatty acid-binding protein aP2 locus reduces the risk for hypertriglyceridemia, type 2 diabetes, and cardiovascular disease. Proc. Natl. Acad. Sci. USA.

[51] Ochoa-Martínez Á.C., Ruíz-Vera T., Pruneda-Álvarez L.G., González-Palomo A.K., Almendarez-Reyna C.I., Pérez-Vázquez F.J., Pérez-Maldonado I.N. (2017). Serum adipocyte-fatty acid binding protein (FABP4) levels in women from Mexico exposed to polycyclic aromatic hydrocarbons (PAHs). Environ. Sci. Pollut. Res. Int..

[52] Ladanchuk T., Kwak S., Bates L., Parkin K., Harris K., Fitzgerald O., Lynch W., Moore K.H. (2018). Vascular measures of atherosclerosis in detrusor overactivity and controls. Neurourol. Urodyn..

[53] Yeniel A.O., Ergenoglu A.M., Meseri R., Kismali E., Ari A., Kavukcu G., Aydin H.H., Ak H., Atay S., Itil I.M. (2018). Is overactive bladder microvasculature disease a component of systemic atheroscleorosis?. Neurourol. Urodyn..

[54] Kershen R.T., Azadzoi K.M., Siroky M.B. (2002). Blood flow, pressure and compliance in the male human bladder. J. Urol..

[55] Tang Y., Donnelly K.C., Tiffany-Castiglioni E., Mumtaz M.M. (2003). Neurotoxicity of polycyclic aromatic hydrocarbons and simple chemical mixtures. J. Toxicol. Environ. Health A.

[56] Wormley D.D., Ramesh A., Hood D.B. (2004). Environmental contaminant-mixture effects on CNS development, plasticity, and behavior. Toxicol. Appl. Pharmacol..

[57] Chepelev N.L., Moffat I.D., Bowers W.J., Yauk C.L. (2015). Neurotoxicity may be an overlooked consequence of benzo[a]pyrene exposure that is relevant to human health risk assessment. Mutat. Res. Rev. Mutat. Res..

[58] Jett D.A., Navoa R.V., Lyons M.A. (1999). Additive inhibitory action of chlorpyrifos and polycyclic aromatic hydrocarbons on acetylcholinesterase activity in vitro. Toxicol. Lett..

[59] Lionetto M.G., Caricato R., Calisi A., Giordano M.E., Schettino T. (2013). Acetylcholinesterase as a biomarker in environmental and occupational medicine: New insights and future perspectives. Biomed. Res. Int..

[60] Kang J.J., Fang H.W. (1997). Polycyclic aromatic hydrocarbons inhibit the activity of acetylcholinesterase purified from electric eel. Biochem. Biophys. Res. Commun..

[61] Fowler C.J., Griffiths D., De Groat W.C. (2008). The neural control of micturition. Nat. Rev. Neurosci..

[62] Li Z., Romanoff L.C., Lewin M.D., Porter E.N., Trinidad D.A., Needham L.L., Patterson D.G., Sjodin A. (2010). Variability of urinary concentrations of polycyclic aromatic hydrocarbon metabolite in general population and comparison of spot, first-morning, and 24-h void sampling. J. Expo. Sci. Environ. Epidemiol..

